# Sporotrichoid Nocardiosis in an Immunocompetent Patient: A Case Report

**DOI:** 10.7759/cureus.75453

**Published:** 2024-12-10

**Authors:** Ivonne De La Hoz, Gabriela De La Hoz, Gurdeep Singh, Manoucher Manoucheri

**Affiliations:** 1 Internal Medicine, AdventHealth Orlando, Orlando, USA

**Keywords:** cutaneous nocardia infection, nocardia, nocardia in immunocompetent, sporotrichoid nocardiosis, sporotrichosis

## Abstract

*Nocardia* spp. rarely cause infection in humans and are most common in the immunocompromised population. Pulmonary nocardiosis is the most common presentation. Cutaneous involvement is usually acquired after direct contact.

A 71-year-old healthy male patient presented with a non-healing wound in his left leg that had appeared two weeks before. The patient had been engaged in yard work after a hurricane. He developed pain in the left calf, later noticing a small wound attributed to an insect bite or a thorn prick. The lesion exhibited increasing surrounding erythema, purulent discharge, and escalating pain. He completed a course of doxycycline prescribed by his primary care physician, but it did not lead to any improvement. The physical examination revealed swelling in the left leg with an ulcer surrounded by erythema. A combination of linezolid and ciprofloxacin was initiated without improvement. Shortly after, lymphangitis was noted, raising concern for sporotrichosis. Antifungal treatment with itraconazole was empirically initiated while continuing linezolid and ciprofloxacin for suspected superimposed bacterial infection. A wound culture revealed beaded gram-positive rods, which were subsequently identified as *Nocardia brasiliensis*. The previous antibiotic regimen was discontinued, and trimethoprim/sulfamethoxazole was initiated; he completed a 12-week course and recovered successfully.

Nocardiosis remains a rare entity and is even rarer in the immunocompetent, in which cutaneous presentations are more common than pulmonary or disseminated forms. This resonates with the case presented who was a healthy male patient. Cutaneous nocardiosis clinical presentation varies widely, the case presented progressed to lymphocutaneous involvement, and it resembled sporotrichosis. The indolent course and the lack of response to traditional therapies suggested a different and rare etiology in our case. Results of cultures usually take several days as *Nocardia* species are slow-growing organisms, which can lead to a delay in diagnosis. Cotrimoxazole monotherapy is the first-line treatment in cutaneous presentations.

## Introduction

*Nocardia* spp. are aerobic gram-positive bacteria that can be found in the soil and rarely cause infection in humans, most commonly in the immunocompromised population. Pulmonary nocardiosis is the most common presentation. Contiguous invasion or hematogenous dissemination can lead to extrapulmonary involvement (pleura, pericardium, and central nervous system) typically in the form of abscesses. Cutaneous involvement usually is acquired after direct contact [1,2]. The following description pertains to an immunocompetent male patient who exhibits a peculiar manifestation of cutaneous nocardiosis.

## Case presentation

A 71-year-old male patient with a past medical history notable only for hypothyroidism presented to the emergency department with a non-healing wound in his left lower extremity that had appeared two weeks before. The patient denied any recent travel history. The patient had been engaged in yard work within his property after a hurricane and then started experiencing pain in the left calf, later noticing a small wound attributed to an insect bite or a thorn prick. The lesion evolved into a pustule that he instinctively popped. Rather than improvement, the lesion exhibited increasing surrounding erythema, purulent discharge, and escalating pain. He sought medical attention and was prescribed a course of doxycycline that he completed without any improvement.

**Figure 1:**
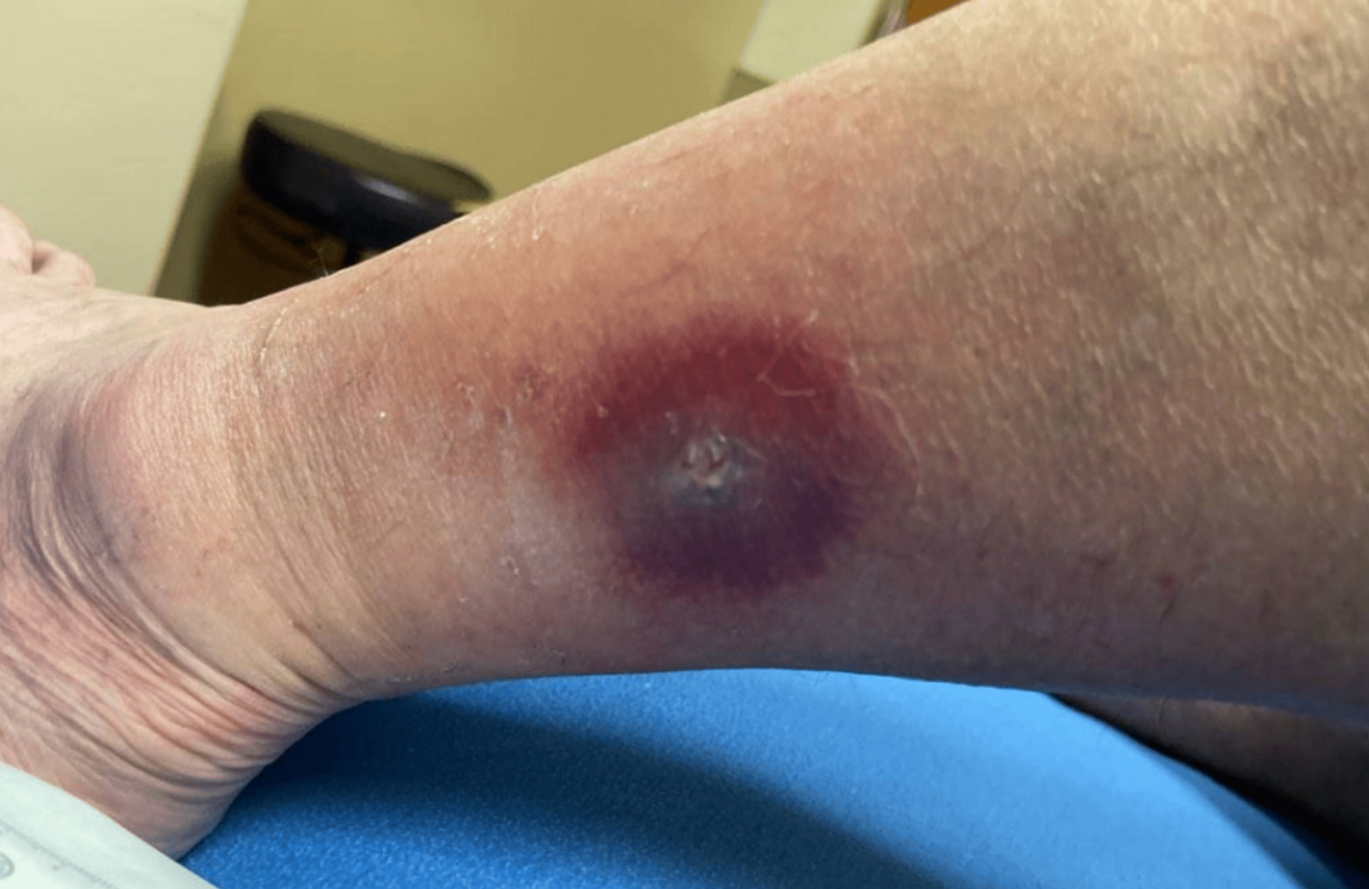
Cutaneous nocardiosis on initial presentation. Note the area of induration with the central ulcer and surrounding erythema.

Upon admission, he was afebrile and hemodynamically stable. The physical examination revealed swelling in the left leg with an ulcer surrounded by erythema (Figure 1). Laboratory results were grossly normal; no leukocytosis or elevation of inflammatory markers was noted (Table 1).

**Table 1:** Laboratory results on admission. BUN: blood urea nitrogen

Component (reference range)	Day 0
Sodium (135-145 mmol/L)	141
Potassium (3.5-5 mmol/L)	4.3
Chloride (98-110 mmol/L)	105
Bicarbonate (24-32 mmol/L)	25
BUN (5-25 mg/dL)	22
Creatinine (0.6-1.2 mg/dL)	0.86
WBC (4.40-10.50 × 10^3^/μL)	6.29
Hemoglobin (12.6-16.7 mg/dL)	15.5
Platelet (139-361 × 10^3^/μL)	178
CRP (<5.00 mg/L)	<3.00

**Figure 2:**
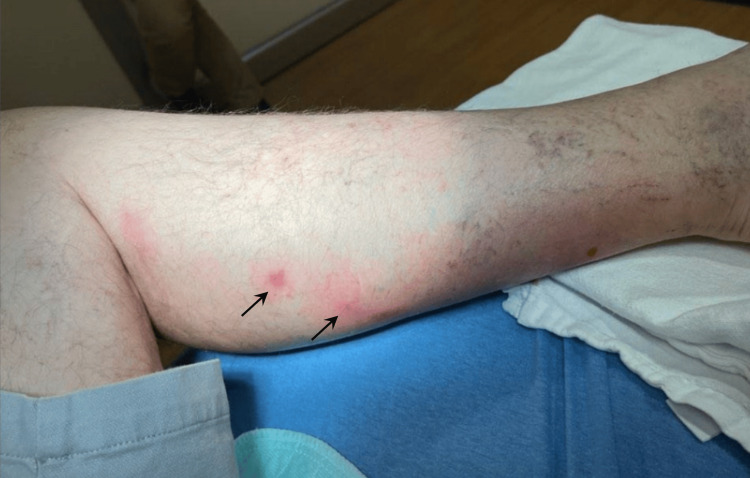
Lymphangitis noted in the left calf (arrows).

Initially, a combination of linezolid and ciprofloxacin was initiated without improvement. Shortly after, lymphangitis was noted (Figure 2), with several new nodules extending proximally and raising concern for sporotrichosis.

**Figure 3:**
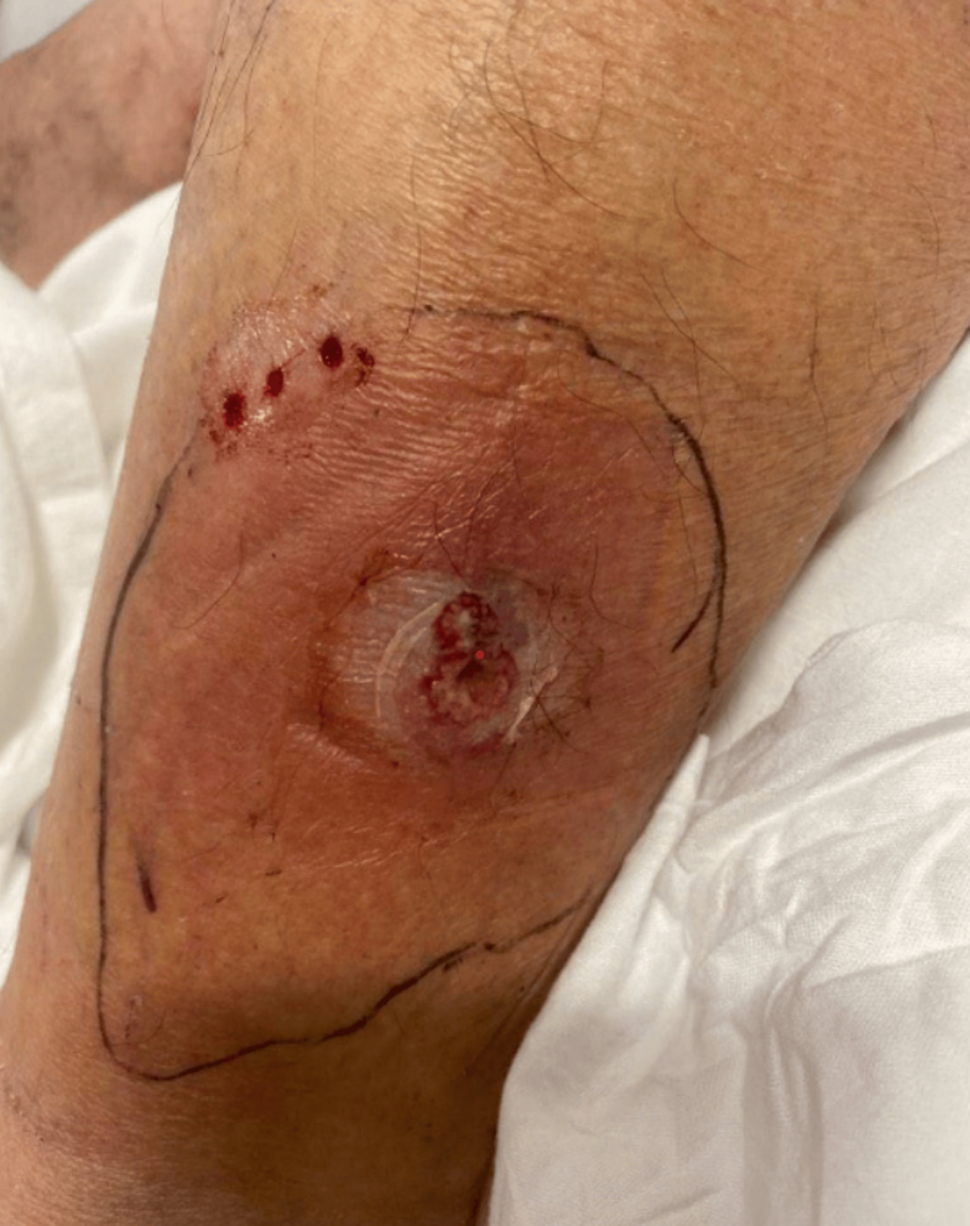
Cutaneous nocardiosis on hospital day 5. Notice the decreased erythema and swelling while on empiric regimen of linezolid, ciprofloxacin, and itraconazole.

Therefore, antifungal treatment with itraconazole was empirically initiated while continuing linezolid and ciprofloxacin for suspected superimposed bacterial infection. Improvement was subsequently noted (Figure 3).

The initial wound culture was negative, but a subsequent culture collected two days later revealed beaded gram-positive rods after 72 hours. Consequently, the previous regimen was discontinued, and trimethoprim/sulfamethoxazole was initiated. Approximately two weeks later, *Nocardia brasiliensis* was identified in the culture of the wound. The patient completed a 12-week course of trimethoprim/sulfamethoxazole and recovered successfully.

## Discussion

Nocardiosis has traditionally been regarded as an opportunistic infection affecting the immunocompromised. Nocardiosis remains a rare entity and is even rarer in the immunocompetent, in which cutaneous presentations are more common than pulmonary or disseminated forms [3]. This resonates with the case presented who was a healthy middle-aged man with no significant underlying diseases. Cutaneous nocardiosis results after direct inoculation, and presentation varies widely and can be in the form of cellulitis, mycetoma, or lymphocutaneous involvement [2].

Frequently, it mimics common conditions such as staphylococcal or streptococcal skin infections, which can be misleading in many cases. The case presented progressed to lymphocutaneous involvement, and it resembled sporotrichosis, commonly referred to as the rose gardener disease, given the higher risk of this group due to frequent soil exposure [4]. Lymphocutaneous nocardiosis is also called sporotrichoid nocardiosis, owing to similarities between both entities as exposed before, and both etiologies should be considered in the right clinical context [2].

Pipito and Cascio presented a comparable case, a 75-year-old female patient who developed tender nodules on the dorsum of the hand, extending to the forearm with axillary lymphadenopathy [5]. Similarly, Dumic et al. reported a case of a 66-year-old female patient without comorbidities who presented with nodular lesions on her left index finger and hand, she was treated with various antibiotic regimens without improvement, and she was diagnosed with nocardiosis [3]. Both cases cited have in common a recent history of injuries sustained while gardening, which was also consistent with the reported history of our patient.

*Mycobacterium marinum* can cause a similar clinical picture; however, it is classically associated with exposure to water environments, which was absent in this case. Similarly, ulceroglandular tularemia, caused by *Francisella tularensis*, presents with comparable features. Human infection occurs through direct contact with rabbits or rodents, or indirect exposure via tick bites. In addition to lymphocutaneous manifestations, systemic symptoms are prominent. *Leishmania braziliensis* is another potential cause of cutaneous and nodular lymphangitis, particularly in Central and South America; however, it is an unlikely diagnosis in this case given the lack of recent travel [6].

The indolent course and the lack of response to traditional therapies suggested a different and rare etiology in our case. Diagnosis is established through microbiological confirmation; in gram and modified acid-fast staining, *Nocardia* spp. present as branching, filamentous, beaded gram-positive bacilli. Several species have been identified as capable of causing cutaneous disease, although *N. brasiliensis* has been associated with lymphocutaneous presentations [4,7]. Results of cultures usually take several days as Nocardia species are slow-growing organisms, which can consequently lead to a delay in diagnosis [7].

Multidrug regimens are required in case of severe pulmonary or disseminated disease. Cotrimoxazole monotherapy is the first-line treatment in cutaneous presentations caused by *Nocardia*. Linezolid is also considered an acceptable alternative [8]. In our case, the patient was already taking linezolid when the antifungal treatment began. This coincided with an improvement that indicated fungal etiology. However, in retrospect, the observed improvement was due to linezolid, which is also effective against *Nocardia*. A 12-week course of trimethoprim/sulfamethoxazole resulted in complete resolution.

## Conclusions

This case highlights the critical importance of obtaining a thorough medical history and maintaining a strong clinical suspicion for nocardiosis early in the course, especially in patients with recent exposure to soil. It also underscores the dangers of anchoring bias, where early assumptions may delay the accurate identification and treatment of uncommon but serious infections. Notably, there is a lack of comprehensive data on cutaneous nocardiosis in medical literature. Large prospective or retrospective reviews have not been conducted in the United States, and the available data consists of sporadic case reports.
